# Social and spatial heterogeneity in psychosis proneness in a multilevel case–prodrome–control study

**DOI:** 10.1111/acps.12384

**Published:** 2014-12-31

**Authors:** J. B. Kirkbride, J. Stochl, J. Zimbrón, C. M. Crane, A. Metastasio, E. Aguilar, R. Webster, S. Theegala, N. Kabacs, P. B. Jones, J. Perez

**Affiliations:** ^1^ Division of PsychiatryUCLLondonUK; ^2^ Department of PsychiatryUniversity of CambridgeCambridgeUK; ^3^ Cambridgeshire & Peterborough NHS Foundation TrustCambridgeUK; ^4^ Department of Health SciencesUniversity of YorkYorkUK; ^5^ Norfolk & Suffolk Foundation TrustIpswichUK; ^6^ Department of Mental HealthParc Tauli Sabadell University HospitalBarcelonaSpain

**Keywords:** psychotic disorders, social environment, epidemiology, prodromal symptoms, population spatial distribution

## Abstract

**Objective:**

To test whether spatial and social neighbourhood patterning of people at ultra‐high risk (UHR) of psychosis differs from first‐episode psychosis (FEP) participants or controls and to determine whether exposure to different social environments is evident before disorder onset.

**Method:**

We tested differences in the spatial distributions of representative samples of FEP, UHR and control participants and fitted two‐level multinomial logistic regression models, adjusted for individual‐level covariates, to examine group differences in neighbourhood‐level characteristics.

**Results:**

The spatial distribution of controls (*n* = 41) differed from UHR (*n* = 48; *P* = 0.04) and FEP participants (*n* = 159; *P* = 0.01), whose distribution was similar (*P* = 0.17). Risk in FEP and UHR groups was associated with the same neighbourhood‐level exposures: proportion of single‐parent households [FEP adjusted odds ratio (aOR): 1.56 95% CI: 1.00–2.45; UHR aOR: 1.59; 95% CI: 0.99–2.57], ethnic diversity (FEP aOR: 1.27; 95% CI: 1.02–1.58; UHR aOR: 1.28; 95% CI: 1.00–1.63) and multiple deprivation (FEP aOR: 0.88; 95% CI: 0.78–1.00; UHR aOR: 0.86; 95% CI: 0.76–0.99).

**Conclusion:**

Similar neighbourhood‐level exposures predicted UHR and FEP risk, whose residential patterning was closer to each other's than controls. Adverse social environments are associated with psychosis before FEP onset.

**Significant outcomes**

- The spatial distribution of controls differed from ultra‐high risk (UHR) and first‐episode psychosis (FEP) participants, who did not differ from each other.
- The same neighbourhood‐level social environmental exposures predicted elevated risk in both the UHR and FEP groups relative to controls, to a similar extent.
- The spatial patterning of FEP is unlikely to be solely due to social drift following the onset of disorder.

**Limitations**

- This multilevel study used cross‐sectional data to compare social and spatial differences in the three groups in a defined catchment area; we did not have longitudinal data on transition to psychosis in UHR participants.
- Controls were broadly similar to the population at risk in sociodemographic terms but came from more densely populated neighbourhoods, making odds ratios conservative.
- We had a relatively small sample of controls and UHR participants in this study.

## Introduction

The incidence of schizophrenia and other non‐affective psychotic disorders is elevated in more densely populated urban areas 1, 2, 3, often characterised by greater social and economic disadvantage 4, 5, 6, 7, 8, 9, 10. Evidence that urban birth and childhood upbringing increase schizophrenia risk in adulthood is consistent with an aetiological role for environment factors 8, 9, 10, although downward social drift of people in their first episode of psychosis (FEP) into lower socio‐economic positions or communities, as a consequence of disorder, has not been entirely refuted. As both causal and consequential factors may explain a degree of the social and spatial patterning of schizophrenia, further investigation of their respective roles is putatively important for both prevention and management of clinical services for people with FEP. Here, careful examination of the social epidemiology of people who meet ultra‐high risk (UHR) criteria for psychosis (due to familial risk and/or early prodromal criteria) may be informative, as this group do not meet diagnostic threshold for FEP. At the individual level, greater psychosocial stress 11, lower social support 11, childhood trauma 12, 13 and receipt of welfare benefits 14 are reported to predict UHR status, in line with similar risk factors for psychotic disorders. Less research has focussed the role of the wider social environment in relation to UHR status. One study observed that urban living was associated with greater risk of transition in a UHR sample 14, although another did not 15. No study has, however, compared the spatial distribution and detailed characteristics of the social environment amongst people with FEP, UHR and population‐based control subjects in a single epidemiological sample, which forms the focus of the present investigation.

### Aims of the study

We hypothesised that people with first‐episode psychosis would have a different social and spatial distribution to controls, towards more socially disadvantaged communities, and that this would be stronger for non‐affective psychotic disorders in line with previous literature; affective psychoses do not appear to vary by urbanicity. We also hypothesised that the sociospatial distribution of the ultra‐high risk group would differ from controls, in similar ways to people with first‐episode psychosis, which together would support the possibility that associations between psychotic disorders and the social environment cannot solely be attributable to social drift following onset.

## Material and methods

### Study design and setting

We used a cross‐sectional study design to identify all incidence cases of FEP, a sample of people meeting UHR criteria for subthreshold psychosis and population‐based controls in the defined catchment area of the Cambridgeshire & Peterborough NHS Foundation Trust (CPFT) in the East of England, UK, over a 20‐month period.

### Participants with first‐episode psychosis

All people with suspected FEP referred to the CAMEO early intervention in psychosis service (EIS) were potentially eligible for inclusion. Participants were identified via the Social Epidemiology of Psychoses in East Anglia (SEPEA) study 16, a larger study of all FEP contacts presenting to six EIS in East Anglia, aged 16–35 years, over 3·5 years. To ensure consistency with the UHR group, we restricted the sample to people first referred between 1 February 2010 and 30 September 2012. Inclusion criteria were as follows:

- Presence of psychotic symptoms at acceptance into EIS care.
- No previous referral to mental health services for psychotic symptoms or treatment with antipsychotic medication.
- Aged 18–35 years (to correspond with control age range, see below).
- Resident within the catchment area at referral.
- Absence of acute intoxication due to substance abuse or withdrawal, an organic basis to presentation or severe learning difficulty (defined by a Weschler Adult Intelligence Scale IQ score <70).

Six months after EIS acceptance, or discharge from the service (whichever was sooner), a research‐based diagnosis was obtained using the operationalised criteria checklist (OPCRIT) 17, a reliable 17, 18 and validated 19 90 symptom‐item assessment for establishing psychiatric diagnoses based on case note review. Participants who met criteria for International Classification of Diseases, Tenth Revision (ICD‐10) F20–33 psychotic disorders were included, with non‐affective (F20–29) and affective psychotic disorder (F30–33) also treated as separate subgroups for analyses. Raters first received OPCRIT training, rating the same set of 12 anonymous case vignettes (not participants in the present study) to establish reliability; formal inter‐rater reliability statistics could not be estimated on 12 vignettes, but percentage agreement ranged from 83% to 100%, based on a comparison of ICD‐10 non‐affective psychosis (F20–29), affective psychosis (F30–33) or not psychotic (data available from authors).

### Participants at ultra‐high risk for psychosis

People meeting UHR criteria for psychosis were identified as part of the PAATh study 20, which ran in parallel to the SEPEA study in CAMEO. All people referred to CAMEO were screened according to the Comprehensive Assessment of At‐Risk Mental States (CAARMS) 21, a clinical instrument with good reliability and valid criteria for the identification of UHR individuals. Inclusion criteria were identical to those for people with FEP, except that they met CAARMS criteria for either.

- Attenuated psychosis (subthreshold symptom intensity or frequency), or
- Brief Limited Intermittent Psychotic Symptoms (threshold symptoms lasting no longer than one continuous week in the last year and having not persisted for over five years), or
- A family history of psychosis in a first‐degree relative or schizotypal personality disorder in the proband, plus a 30% drop in Global Assessment of Functioning (GAF) score from premorbid level, sustained for a month, within the past 12 months, or GAF score of 50% or less for the past 12 months.

### Control participants

Controls, aged 18–35 years old, without psychosis, were identified from an embedded project within the SEPEA study, the European Union Gene‐Environment Interaction (EU‐GEI) study. This is an ongoing international, multi‐centre case–sibling–control study of gene–environment interactions in schizophrenia and other psychoses in people aged 18–64 years 22. Controls were identified via multistage, stratified random sampling to ensure representativeness to the population at risk. First, a sampling frame of all general practices (GPs) (*N* = 103) within the CPFT catchment area was established, from which we randomly invited 20 practices to participate. Due to refusal (*N* = 14), we resampled 20 further practices, without replacement, until ten practices had been enrolled. Second, GP patient lists were screened (by the practice manager) to exclude people who did not meet inclusion criteria (as above, but not meeting FEP or UHR criteria). GPs also retained the right to remove any patients deemed inappropriate for contact (such as recently bereaved individuals), which was minimal in practice. From each patient list, we randomly invited 150 participants to take part in the EU‐GEI study, contacted by letter and follow‐up phone calls. Participants who responded positively were recruited for full EU‐GEI assessment until an *a priori* target (*n* = 105) had been achieved.

### Individual sociodemographic variables

We collected baseline sociodemographic data on participants at first contact, including age, sex, ethnic group, main or last occupation and residential postcode. Self‐ascribed ethnicity to one of 18 major ethnic groups, as per the 2011 Census of Great Britain 23, was collapsed for analysis into a six‐category variable (white British, white other, black, Asian, mixed ethnicities, other) and a binary variable [white British, all black and minority ethnic (BME) groups]. Main occupation was coded to the National Statistics Socioeconomic Classification (NS‐SEC) 6‐category socioeconomic variable, adhering to strict decision rules 24: professional and managerial occupations, intermediate and self‐employed, semi‐routine and routine, students, long‐run unemployed, otherwise unclassifiable. Highest main (or if not available, current, or last) parental occupation (fathers' or mothers') was coded similarly.

### Neighbourhood‐level socioenvironmental exposures

We geocoded each participant's residential postcode to their corresponding latitude/longitude coordinates (British National Grid) to (i) examine differences in the spatial distribution of the three groups, and (ii) assign participants to their neighbourhood of residence, delimited here by 2011 census wards [*N* = 150 wards, median population: 4984; interquartile range (IQR): 2761–7430]. For each ward (henceforth the ‘neighbourhood’), we measured *a priori* socioenvironmental exposures, estimated from the 2011 census (see Table 1 for full details): neighbourhood‐level population density (people per hectare), proportion of households deprived on at least two of four 2011 census deprivation domains 25, inequality in multiple deprivation between output areas (OA) (median population: 311; IQR: 267–353) nested within each neighbourhood 7, proportion of single‐person households, proportion of single‐parent households, proportion of people aged 18–35 years old (as a marker of social isolation from other young people), own‐group ethnic density (estimated for the six ethnic groups), own‐group ethnic segregation (the extent to which each ethnic group was concentrated at OA‐level within each neighbourhood) 6 and ethnic diversity [a measure borrowed from ecology to estimate diversity 26].

**Table 1:** Overview of included neighbourhood‐level (electoral ward) socioenvironmental exposure variables*

Variable	Source(s)	Description
Population density	Table QS103EW 2011 Census	People per hectare, estimated from usual resident population size divided by ward area
% multiple deprivation	Table QS119EW 2011 Census	Proportion of households classified as meeting criteria for deprivation on at least two of four Census domains (employment, education, health and housing quality). See 25 for full details of Census methodology
% inequality in multiple deprivation	Table QS119EW 2011 Census	Estimate of disparity in *% multiple deprivation* at smaller geographical level (Output Area) within each electoral ward. Calculated using Gini coefficient and expressed as a proportion (0 = no inequality, 100 = perfect inequality)
% single‐person households	Table KS105EW 2011 Census	Proportion of single‐person households as a total of all households per ward
% single‐parent households	Table KS105EW 2011 Census	Proportion of single‐parent households with dependent children as a total of all households per ward
% people aged 18–35 years	Table QS103EW 2011 Census	Proportion of the total population per ward aged 18–35 years old as a marker of social isolation amongst young people
% own‐group ethnic density	Table DC2101EW Census 2011	Proportion of total population per ward belonging to each given ethnic group
% own‐group ethnic segregation	Table DC2101EW Census 2011	Extent to which each ethnic group was concentrated or dispersed across each ward [at output areas (OA)‐level], relative to all other groups. Estimated using Index of Dissimilarity 7. (0 = total integration, 100 = total segregation)
% ethnic diversity	Table KS201EW Census 2011	Measure borrowed from ecology to estimate diversity 26, defined by the reciprocal diversity index, which here estimates the total number ethnic groups in a neighbourhood (*N*_max_ = 18), weighted by their population size; it may range from 1 to *N*_max_ and is rescaled as a proportion (0 = maximum ethnic homogeneity, 100 = maximum ethnic diversity)

*Variables estimated from the Office for National Statistic's 2011 Census of Great Britain 23.

### Statistical methods

Prior to our main statistical analysis, we assessed whether our final sample of control participants was representative of the wider population at risk by comparing them to excluded participants and the population at risk in the catchment area estimated from the 2011 Census of Great Britain 23 on a range of individual and neighbourhood‐level exposures. Chi‐squared tests and Fisher's exact tests were used to compare categorical variables (sex, ethnicity, socioeconomic status); Mann–Whitney *U*‐tests were used to test median differences in continuous variables (age, neighbourhood variables). Differences in individual‐level exposures between FEP, UHR and control participants were assessed similarly.

We next inspected whether the spatial distribution of people with FEP, the UHR group and controls differed at first contact using a two‐dimensional *M*‐test 27, 28. This assesses whether the distribution of interpoint distances between all observations from two participant groups (i.e. FEP vs. controls, FEP vs. UHR participants, etc.) differs in a two‐dimensional space (i.e. latitude/longitude); the null hypothesis is that the two groups come from the same spatial distribution. Next, we inspected whether any neighbourhood‐level socioenvironmental exposures might be associated with such differences by fitting two‐level (individuals nested in neighbourhoods), multinomial logistic regression models. Multinomial models allow for the simultaneous estimation of risk (i.e. odds) in the UHR and FEP group relative to controls. By extending these models to a multilevel framework, we can estimate unmeasured variation in psychosis proneness attributable to neighbourhood effects, via inclusion of a latent random effect. We had no reason to assume the neighbourhood would have different (random) effects on UHR or FEP risk and so fitted a single random intercept which could vary between neighbourhoods, but was constrained to have the same effect across groups.

A null model (without covariates or ‘fixed’ effects) was first fitted to quantify variation in psychosis proneness attributable to neighbourhood random effects. Next, we entered neighbourhood‐level fixed effects one‐by‐one into univariable models to examine their association with psychosis proneness (control vs. UHR vs. FEP). Model fit was assessed via Akaike's Information Criterion (AIC), where lower scores indicated better fit. We then employed a backward‐fitting modelling strategy to identify our best‐fitting model, having included all individual variables as potential *a priori* confounders. All neighbourhood‐level variables were tested sequentially (in order of poorest AIC from univariable analyses) for removal from the model, assessed via a permissive likelihood ratio test criteria of *P* < 0.10. To examine whether non‐affective and affective psychotic disorders differed with respect to the environment, we refitted our final model with these disorders as separate multinomial outcomes. We reported odds ratios (OR) and 95% confidence intervals (95% CI). Modelling was conducted in stata (version 13, StataCorp, College Station, TX, USA), with two‐level multinomial logistic regressions fitted via generalised structural equation models (*gsem*).

### Ethics committee approval

The SEPEA, PAATh and EU‐GEI studies received full ethical approval from the Cambridgeshire East Research Ethics Committee.

## Results

### Sample representativeness

One‐hundred and eighty‐nine people, aged 18–35 years, with potential FEP were accepted by CAMEO over the study period, of whom 22 were excluded because of the absence of an OPCRIT‐confirmed ICD‐10 diagnosis. A further eight clients of no fixed abode were also excluded, leaving 159 people with FEP in the present analyses, of which 131 (82.3%) received an ICD‐10 diagnosis of non‐affective psychosis. Excluded FEP participants did not differ by median age (Mann–Whitney *U*‐test *P* = 0.21), sex (χ^2^‐test *P* = 0.74), ethnic status (white British vs. BME: χ^2^‐test *P* = 0.96), marital status (χ^2^‐test *P* = 0.69), or highest participant (Fisher's Exact test *P* = 0.94) or parental (Fisher's Exact test *P* = 0.30) socioeconomic status. Forty‐nine people met UHR criteria for psychosis (all CAARMS criteria 1), of whom one participant living outside of the catchment area at first contact, was excluded. Forty‐one EU‐GEI controls, aged 18–35 years, were included in this analysis.

Controls were representative of the population at risk from the 2011 census in terms of available sociodemographic data on age group (Fisher' Exact test *P* = 0.76), sex (χ^2^‐test *P* = 0.54), white British vs. BME ethnicity (χ^2^‐test *P* = 0.83) and socioeconomic status (Fisher's Exact test *P* = 0.34). Data on marital status and parental socioeconomic status could not be compared as these were not available from the 2011 Census for the population at risk aged 18–35 years. Control neighbourhoods were representative of the wider CPFT catchment area in terms of median deprivation (20.2% vs. 20.7%; Mann–Whitney *U*‐test *P* = 0.15), inequality (23.3% vs. 20.5%; Mann–Whitney *U*‐test *P* = 0.72), single‐parent households (4.8% vs. 4.7%; Mann–Whitney *U*‐test *P* = 0.25) and ethnic segregation (18.2% vs. 19.8% Mann–Whitney *U*‐test *P* = 0.90), but were more densely populated (11 people per hectare vs. 2.1; Mann–Whitney *U*‐test *P* = 0.06), ethnically diverse (2.7% vs. 0.7%; Mann–Whitney *U*‐test *P* = 0.002) and had higher proportions of people aged 18–35 years (24.5% vs. 16.8%; Mann–Whitney *U*‐test *P* = 0.004) and single‐person households (28.7% vs. 26.3%; Mann–Whitney *U*‐test *P* = 0.03).

### Participant characteristics

Controls were significantly older than FEP (*P* = 0.02) or UHR participants (*P* < 0.001) (Table 2). We observed a trend for greater proportions of men with increased psychosis proneness (*P* = 0.06), while controls held higher socioeconomic occupations than their UHR or FEP counterparts (*P* = 0.003). Differences in highest parental socioeconomic occupations were also apparent (*P* = 0.04), with parents of UHR participants somewhat over‐represented in professional and managerial occupations.

**Table 2:** Clinical and sociodemographic sample characteristics, by participant status

Variable	People with FEP	UHR group	Controls	*P*‐value*
Median age (IQR)	24.3 (21.3, 29.0)	20.5 (18.9, 22.8)	27.0 (23.0–32.0)	FEP vs. UHR: *P* < 0.001 FEP vs. control: *P* = 0.02 UHR vs. control: *P* < 0.001
Total participants (*N* = 248)	159	48	41	–
Men (*N*, %)	101 (63.5)	24 (50.0)	19 (46.3)	0.06^C^
White British (*N*, %)	100 (62.9)	44 (91.7)	30 (73.2)	<0.001^C^
Single† (*N*, %)	139 (87.4)	41 (85.4)	31 (75.6)	0.17^C^
Socioeconomic status (*N*, %)				
Professional and managerial	18 (11.3)	6 (12.5)	15 (36.6)	0.003^FE^
Intermediate and self‐employed	23 (14.5)	6 (12.5)	9 (22.0)	
Semi‐routine and routine	70 (44.0)	17 (35.4)	11 (26.8)	
Students	28 (17.6)	10 (20.8)	5 (12.2)	
LR unemployed	17 (10.7)	5 (10.4)	–	
Unclassifiable	3 (1.9)	4 (8.3)	1 (2.4)	
Parental socioeconomic status (*N*, %)				
Professional and managerial	47 (29.6)	26 (54.2)	17 (41.5)	0.04^FE^
Intermediate and self‐employed	34 (21.4)	10 (20.8)	11 (26.8)	
Routine and manual	46 (28.9)	6 (12.5)	10 (24.4)	
Students	2 (1.3)	–	–	
LR unemployed	9 (5.7)	4 (8.3)	–	
Unclassifiable	21 (13.2)	2 (4.2)	3 (7.3)	
Non‐affective psychosis (F20–29)	131 (82.4)	–	–	–
Affective psychosis (F30–33)	28 (17.6)	–	–	–

FEP, first‐episode psychosis; UHR, ultra‐high risk; IQR, interquartile range.

*We used the Mann–Whitney *U*‐test to inspect differences in median age between each pair of participant groups separately. For categorical variables, we used Chi‐squared tests (superscript: C) or Fisher's exact tests (where any cell *n* ≤ 5; superscript: FE) to inspect differences between the three groups simultaneously.

†Single included all single, widowed and divorced participants at assessment vs. people either married or in a civil partnership.

### Spatial distribution of participants at first contact

**Figure 1:**
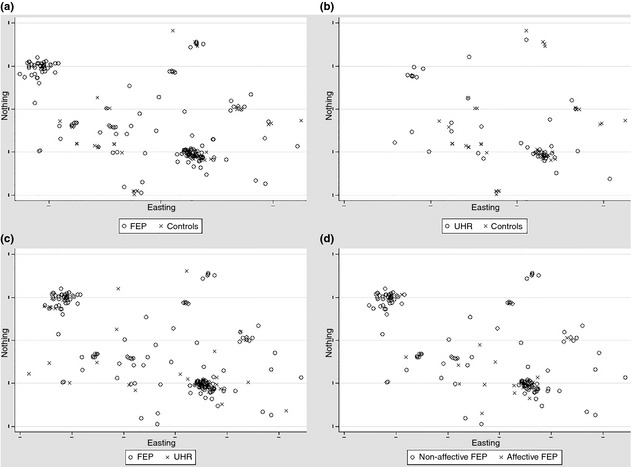
Spatial locations of participants, by status. The spatial distribution of controls is significantly different to both people with (a) first‐episode psychosis (FEP) (*P* = 0.01) and (b) the ultra‐high risk (UHR) group (*P* = 0.04) under a two‐dimensional *M*‐test. There is no statistically significant difference in the spatial distribution of (c) FEP and UHR participants (*P* = 0.17). The spatial distribution of (d) people with non‐affective and affective FEP were also significantly different from each other (*P* = 0.01). Locations are based on postcode centroid at first contact. Axis scales are plotted according to British National Grid coordinates of residential postcode at first contact, but the coordinates and scale have been removed to preserve sample anonymity.

The spatial distribution of controls differed from both the FEP (*P* = 0.01) and UHR groups (*M*‐test *P* = 0.04) (Fig. 1a,b). There was no evidence that the spatial distribution of the FEP group differed from the UHR group (*P* = 0.17; Fig. 1c). These patterns held when the analyses were restricted to people with non‐affective FEP (*n* = 131; vs. controls: *P* = 0.01; vs. UHR: *P* = 0.22) and affective psychoses (*n* = 28; vs. controls: *P* = 0.01; vs. UHR: *P* = 0.62), whose spatial distributions also differed significantly from each other (*P* = 0.01; Fig. 1d).

### Multilevel multinomial regression

A null multilevel model provided some weak evidence that the risk of psychosis proneness varied at the neighbourhood level (*P* = 0.07; Table 3). Following model building, we observed that the adjusted odds of UHR or FEP status, relative to controls, were similarly elevated in neighbourhoods characterised by greater proportions of single‐parent households, lower deprivation and greater ethnic diversity (Table 3). For example, a one per cent increase in the proportion of single‐parent households was associated with an increased adjusted odds ratio (aOR) of UHR status of 1.59 (95% CI: 0.99, 2.57; *P* = 0.056) and FEP status of 1.56 (95% CI: 1.00, 2.45; *P* = 0.052). The odds of UHR (aOR: 0.86; 95% CI: 0.76, 0.99; *P* = 0.033) and FEP status (aOR: 0.88; 95% CI: 0.78, 1.00; *P* = 0.046) also independently *decreased* with greater neighbourhood‐level deprivation in our final model, while greater ethnic diversity increased the odds of membership in either the UHR (aOR: 1.28; 95% CI: 1.00, 1.64; *P* = 0.046) or FEP group (aOR: 1.28; 95% CI: 1.02, 1.59; *P* = 0.030). Individual‐level socioeconomic status was also independently associated with greater odds of UHR and FEP status. These associations broadly held when non‐affective and affective FEP were modelled as separate multinomial outcomes in the same model (Table 4).

**Table 3:** Adjusted odds of FEP or high‐risk status vs. controls in final two‐level multinomial model associated with individual‐ and neighbourhood‐level exposures

	People with FEP aOR (95% CI)	UHR group aOR (95% CI)
Individual‐level exposures		
Age (years)	0.94 (0.84, 1.07)	0.71 (0.60, 0.84)*
Men (vs. women)	2.03 (0.73, 5.64)	1.31 (0.40, 4.31)
BME status (vs. white British)†	0.72 (0.22, 2.45)	0.19 (0.04, 0.97)*
Single marital status (vs. married)	1.01 (0.25, 4.04)	0.23 (0.04, 1.34)
Socioeconomic status‡	1.79 (1.11, 2.88)*	1.78 (1.03, 3.08)*
Parental socioeconomic status‡	1.25 (0.89, 1.77)	0.95 (0.62, 1.44)
Neighbourhood‐level exposures		
% Single‐parent households	1.56 (1.00, 2.45)*	1.59 (0.99, 2.57)**
% Ethnic diversity	1.28 (1.02, 1.59)*	1.28 (1.00, 1.64)*
% Households in multiple deprivation	0.88 (0.78, 1.00)*	0.86 (0.76, 0.99)*
Neighbourhood‐level random effects	Variance (SE)	Wald *P*‐value
Null model	3.64 (2.03)	0.07
Individual‐adjusted model	3.72 (2.20)	0.09
Fully adjusted model	2.58 (1.62)	0.11

FEP, first‐episode psychosis; UHR, ultra‐high risk; aOR, adjusted odds ratio; CI, confidence interval; BME, black and minority ethnic; SE, standard error.

†Due to the small sample of BME participants, models with a six‐category ethnicity variable would not converge, and so the binary white British vs. BME variable was substituted.

‡aOR associated with one‐category decline in socioeconomic status. LRT *P*‐value suggested a model fitted with social class (participant and parental) categories as categorical indicator variables did not improve fit: *P* = 0·11.

*P < 0.05; **P < 0.10.

**Table 4:** Adjusted odds ratios from re‐fitted final two‐level multinomial model with non‐affective and affective FEP as separate outcomes

	Non‐affective psychosis aOR (95% CI)	Affective psychosis aOR (95% CI)	UHR group aOR (95% CI)
Individual‐level exposures			
Age (years)	0.96 (0.85, 1.09)	0.88 (0.75, 1.03)	0.71 (0.60, 0.84)*
Men (vs. women)	2.36 (0.84, 6.66)	1.13 (0.33, 3.90)	1.26 (0.38, 4.14)
BME status (vs. white British)†	0.65 (0.19, 2.25)	1.13 (0.27, 4.76)	0.20 (0.04, 1.02)**
Single marital status (vs. married)	1.10 (0.27, 4.54)	0.71 (0.12, 4.03)	0.23 (0.04, 1.32)**
Socioeconomic status‡	1.78 (1.10, 2.88)*	1.76 (0.98, 3.15)**	1.80 (1.04, 3.10)*
Parental socioeconomic status‡	1.32 (0.92, 1.86)	0.92 (0.59, 1.44)	0.92 (0.61, 1.41)
Neighbourhood‐level exposures			
% Single‐parent households	1.56 (0.99, 2.44)**	1.55 (0.95, 2.55)**	1.59 (0.99, 2.56)**
% Ethnic diversity	1.27 (1.02, 1.59)*	1.25 (0.98, 1.61)**	1.28 (1.00, 1.64)*
% Households in multiple deprivation	0.90 (0.79, 1.01)**	0.82 (0.71, 0.95)*	0.86 (0.75, 0.98)*

Neighbourhood‐level random effects	Variance (SE)	Wald *P*‐value
Null model	3.91 (2.18)	0.07
Model adjusted for individual effects	3.30 (1.94)	0.09
Fully adjusted model	2.54 (1.61)	0.11

UHR, ultra‐high risk; aOR, adjusted odds ratios; CI, confidence interval; BME, black and minority ethnic; SE, standard error; FEP, first‐episode psychosis.

†Due to the small sample of BME participants, models with a six‐category ethnicity variable would not converge, and so the binary white British vs. BME variable was substituted.

‡aOR associated with one‐category decline in socioeconomic status.

*P < 0.05; **P < 0.10.

## Discussion

### Key results

To our knowledge, this is the first study to have explored geographical and social differences in residential environments between people with FEP, UHR participants and a representative sample of controls. The spatial distribution of both the UHR and FEP group differed from controls, and for FEP participants, these differences were apparent for non‐affective and affective psychotic disorders independently. This latter finding was unexpected given previous literature suggests an absence of neighbourhood‐level variation in affective psychoses 1, 29. Although the spatial patterning of non‐affective and affective psychotic disorders also differed from each other, risk appeared to be associated with the same set of neighbourhood‐level social exposures. These factors, which included the proportion of single‐parent households, deprivation and ethnic diversity, similarly predicted UHR status, raising the possibility that exposure to adverse environments may affect the population expression of psychosis from subthreshold UHR criteria through to full psychotic disorder. Interestingly, these neighbourhood‐level trends persisted despite control for several important individual‐level confounders, including parental and participant socioeconomic position, ethnicity, age and sex.

### Sources of possible bias

Controls were not identified via simple random sampling from across the CPFT catchment area, but by multistage sampling based on GP location. Nevertheless, they were broadly representative of the underlying population at risk in the catchment area on a range of measured covariates, including ethnicity, individual socioeconomic status and neighbourhood‐level deprivation, inequality, ethnic segregation and the proportion of single‐parent households. Although controls from more urban populations were somewhat over‐represented in our sample (indexed by greater population density, non‐white British ethnic density and proportion of single‐person households), incidence studies of FEP have shown that these neighbourhoods increase risk 4, 6, 7, so any bias would have made reported effect sizes conservative. Under *gsem,* we could not use probability weights to account for the multistage sampling design and associated non‐response at each level of selection. However, this was possible via ordinary (i.e. single level) multinomial logistic regression with robust standard errors (adjusted for neighbourhood clustering); after calculating and including inverse probability weights for controls, all neighbourhood‐level exposures remained statistically significant in our final model (data available from authors).

Ultra‐high risk participants were treated according to initial status, regardless of later transition, which occurred in about 10% of participants over 12 months 30. We have previously described individual level correlates of transition in this sample 20, 30.

### Chance and confounding

We had a relatively small sample of control and UHR participants, which may have limited power to detect certain effects, including random effects at the neighbourhood level (for which there was some support). We also had a very small sample of people with affective psychotic disorder, making findings with respect to this subgroup tentative. We did not have data on family history of psychiatric disorder in FEP or control participants, or cannabis use in our sample, two potentially important unmeasured confounders. Neighbourhood exposures were assessed cross‐sectionally, based on residential neighbourhood at first contact; we were unable to assess cumulative exposure to neighbourhood effects which may have accrued over the life course. Our study also had several strengths, including a robust FEP and UHR ascertainment procedure based on epidemiological principles and a tightly defined catchment area, and appropriate use of novel spatial and multilevel multinomial logistic regression models.

### Interpretation

Our results support the possibility that people at UHR of psychosis are exposed to similar adverse neighbourhood conditions prior to transition as people already in their first episode, even after controlling for more direct markers of social disadvantage such as socioeconomic status. Relevant neighbourhood‐level exposures identified in this study were related to both socioeconomic deprivation and social cohesion; the neighbourhood proportion of single‐parent households may have been a proxy for deprivation, given substantial correlation between these exposures (*r *=* *0.66). Having controlled for this, the counterintuitive negative association between multiple deprivation and psychosis proneness might indicate a nonlinear relationship between psychosis and deprivation 31. The observed association between psychosis proneness and greater neighbourhood ethnic diversity may have been a neighbourhood‐level proxy for individual BME status, for which we had a small sample to detect variation in risk, but which is a well‐established risk factor for psychotic disorders 32. It may also have been a proxy for lower neighbourhood‐level ethnic density, given near‐perfect negative correlation between the two (*r *=* *−0.99). Ethnic density attenuates schizophrenia risk in some ethnic groups 33, perhaps via greater social cohesion 6, 34 mediating the deleterious effects of discrimination 35, 36. Interestingly, both lower cohesion and greater discrimination have now been associated with risk of subthreshold psychosis 34, 35 and clinically relevant psychotic disorder 6, 36.

Several studies have demonstrated associations between urban birth, upbringing and later non‐affective psychosis risk 8, 9, 10. These strengthen evidence for a causal relationship between disorder and aspects of the social environment, unless social drift begins in the parental generation (or earlier); to our knowledge, no study has investigated the role of intergenerational social drift, although the over‐representation of parents of UHR participants in our study in the highest socioeconomic category was not consistent with this. An alternative possibility is that UHR participants had already begun to experience social drift earlier in their prodrome; they experienced the biggest difference in social status of all three groups when compared to their parents' occupational position and there is evidence elsewhere that UHR groups already exhibit certain premorbid cognitive deficits 37. Unfortunately, we did not have sufficient longitudinal data on our UHR group to investigate neighbourhood residential changes over time in relation to risk of transition. Future prospective studies are now required to build on the small body of equivocal research on the risk of transition from UHR to FEP in relation to residential social environments 14, 15, in line with similar research which has addressed transition in relation to individual‐level childhood adversities 38.

Our results have potential implications for aetiology, prevention and the provision of mental health services in terms of early intervention and early detection of psychosis. In addition to the concentration of psychosis risk in socially disadvantaged neighbourhoods determined aetiologically, duration of untreated psychosis may also be longer in these communities 39, potentially exacerbating prognosis in already disadvantaged groups. In public health terms, the debate over social drift vs. causation may be relatively sterile, given that our data, and that of others 39, 40, 41, suggest that our most disadvantaged and fragmented communities will shoulder a disproportionate burden of this population‐level psychiatric morbidity, regardless of causality. From a public mental health perspective, identifying which aspects of neighbourhood social inequality increase the risk of transition to FEP or introduce delays to help‐seeking will be important in designing effective early detection services and prevention strategies which target improvements in the long‐term social and clinical outcomes for young people at risk of psychotic disorder.

## Declarations of interest

None of the authors have any conflicts to declare in the past 2 years. A medical writer or editor was not used in the preparation of this manuscript.
